# Supplementary material for: Positive Interventional Effect of Engineered Butyrate-Producing Bacteria on Metabolic Disorders and Intestinal Flora Disruption in Obese Mice
Source: Microbiol Spectr. 2022 Mar 16;10(2):e01147-21. doi: 10.1128/spectrum.01147-21 (PMC9045090; doi:10.1128/spectrum.01147-21)

Supplementary materials

METHODS

The GC-MS method for measuring butyric acid content

For pretreatment, the collected supernatant assay samples were acidified. The supernatant was placed in a 5 mL polyethylene centrifuge tube with 0.4 mL of 50% sulfuric acid and ether, incubated in a shaker at room temperature and 200 rpm for 45 min, then centrifuged at 3000 rpm for 5 min, and the supernatant was removed in another sterile centrifuge tube and dehydrated by adding anhydrous calcium chloride, after which the supernatant was further used for GC-MS analysis.

GC-MS conditions: column Agilent 123-7032 DB-WAX quartz capillary column (30 m x 320 μm x 0.25 μm); ramp-up procedure: column temperature starting at 60 °C, hold for 2 min, ramp up to 220 °C at 10 °C/min, hold for 20 min, helium as carrier gas, flow rate of 1 mL/min. Mass spectrometry conditions: EI ion source 70 eV, ion source temperature 230 °C, quadrupole temperature 150 °C, solvent delay time 2 min, scan mass range m/z 20-150.

Hepatic tissue hematoxylin eosin and oil red O staining

Hematoxylin eosin staining was performed and the isolated liver tissues were taken and cut into 2×3 mm size tissue blocks, after which they were fixed in 4% formalin. The fixed tissues were washed three times with PBS and processed according to the following procedure. Gradient dehydration treatment, 70% ethanol 0.5-1 h, 80% ethanol 0.5 h, 90% ethanol 0.5 h, 95% ethanol 0.5 h, 95% ethanol 0.5 h, anhydrous ethanol 1 h, anhydrous ethanol 0.5 h, anhydrous ethanol 0.5 h. Tissues were transparent and immersed in wax, 100% xylene I for 15 min and 100% xylene II for 15 min, and transferred to melted paraffin I for 1 h and paraffin II for 1 h after transparency. Tissue sections were dewaxed and rehydrated after staining. Hematoxylin staining: Hematoxylin staining for 3-5 min, using 1% hydrochloric acid alcohol fractionation, the color gradually returned to blue, water washing. Eosin staining: the sections were sequentially stained with 50% ethanol for 1 min, 70% ethanol for 10 s, 80% ethanol for 10 s, 95% ethanol for 2 min, anhydrous ethanol for 2

min, anhydrous ethanol for 2 min, and eosin re-staining for 4 min. Dehydration blocking: anhydrous ethanol, xylene (1:1) mixture for 2 min, xylene I for 5 min, xylene II for 5 min. microscopic examination was used and images were acquired for analysis.

The specific steps of Oil Red O staining are as follows. Fresh frozen sections were fixed by rewarming and drying frozen sections, fixed in fixative for 15 min, and then washed with tap water and dried. For oil red staining, sections were stained by immersion in oil red staining solution for 8-10 min. For background differentiation, sections were removed and left for 3 s and then sequentially submerged in 60% isopropanol for 3 s and 5 s each. Sections were sequentially submerged in 2 dips of pure water for 10 s each. For hematoxylin staining, the sections were removed, left for 3 s and then immersed in hematoxylin for 3-5 min, 3 times pure water immersion wash for 5 s, 10 s and 30 s in sequence. differentiation solution (60% alcohol as solvent) for 2-8 s, 2 times distilled water immersion wash for 10 s in sequence, return blue solution for 1 s. The sections were gently immersed in tap water for 2 times immersion wash for 5 s and 10 s each, and the staining effect was examined microscopically. To seal the tablets, glycerin gelatin sealer was used to seal the tablets. Microscopic microscopy, image acquisition and analysis.

SUPPLEMENTAL TABLES

Table S1 Strains and plasmids used in this study

Strain or plasmid	Characteristics	Source
**Strains**		
E. coli		
Trans-DH5α	F-φ80 lac ZΔM15 *Δ*(lacZYA-arg F) U169 endA1 recA1 hsdR17 (rk-,mk+) supE44λ- thi -1 gyrA96 relA1 phoA	TransGen Biotech
B. subtilis		
SCK6	ErmR, 1A751 derivate, *lacA*::P*xylA-comK*	MiaoLing Plasmid Sharing Platform
RS06551	ErmR, 1A751 derivate, *lacA*::P*xylA-comK*, *ΔskfA ΔsdpC ΔacdA ΔackA* :: butyryl-CoA: acetate: CoA transferase	This work
**Plasmids**		
pCas	*repA*101(Ts) *kan* P_{cas}-cas9 P_{araB}-*Red* *lacI*^{q} P_{trc}-*sgRNA-pMB1*	(1)
pTargetF	Harboring sgRNAs, with or without donor DNAs	(1)
pTargetF-P1	*pMB1 aadA* sgRNA-*skfA*	This work
PTargetF-P2	*pMB1 aadA* sgRNA-*sdpC*	This work
pTargetF-P3	*pMB1 aadA sgRNA-acdA*	This work
pTargetF-P4	*pMB1 aadA sgRNA-ackA*	This work
PTargetF-P5	*pMB1 aadA* sgRNA- butyryl-CoA: acetate: CoA transferase	This work

Table S2 Primers and N20 sequence used in this study

Name	Sequence (5'-3')
**Primer**	
ΔskfA-up-S	ACAGCGCTTGCTCTGTTTCCG
ΔskfA-up-A	TTCCCATTCTTTTTGGTTTCTTTTCATAAG
ΔskfA-down-S	CATCCTGCTATGAGAGCTATTTAACATTTGAG
ΔskfA-down-A	TTAAACCCTTTGATCAGCTTTGCTTCAC
ΔsdpC-up-S	AATGAATCTTTCACAAAGAAGACAGTATTG
ΔsdpC-up-A	CAATAGCCTAAGTAATTTACTTTTCAAATTATTATACC
ΔsdpC-down-S	GCAAAAACCCTAAAATTGATTCATCAATAATC
ΔsdpC-down-A	CAGGAAATAAACCGGCTGCAATTGTATC
ΔacdA-up-S	CATCAATAATATCTTCTTCGATCTCAAGATCATC
ΔacdA-up-A	CTGGTCATCTCCAGAATGCTCGCG
ΔacdA-down-S	CTCATGTTCTTCTGATAATGAAAAATTCATTGC
ΔacdA- down-A	CACGCCGCGCCGCCTC
ΔackA-up-S	GATTTCTCTGACCACATGCGCAAGTTC
ΔackA-up-A	GCGCGCGACGTTGTTCGTTTAG
ΔackA-down-S	TCCTGCGTTAATTGCAATAATTTTGGAC
ΔackA- down-S	CAAGCTGAAACAGTTTTTCAAAGATAAGGTTC
BCoAT-S	ATGGATTTTACGGAATTGTATGCGCAG

BCoAT-A	TCAGCGCTTGTTGCTCCTGCG
**N20**	
skfA edition	CTGCTGGCTGCATGGGCTGT
sdpC edition	TTCATTAGTAGGACTCTCTA
acdA edition	TAGAATTCATGGGCGTATAC
ackA edition	CACTCAAGAAATTCAAAGAC
BCoAT edition	CATAATGAACGCAAAAAAAC
**gRNA sequence**	
gRNA scaffold	GTTTTAGAGCTAGAAATAGCAAGTTAAAATAAGGCTAGTCCGTT ATCAACTTGAAAAAGTGGCACCGAGTCGGTGCTTTTTTTG

Table S3

Results of multiplex analysis of species and gene functions at different levels

Table S4

168 annotated compounds peak intensity table

Table S5

Enrichment analysis pathway annotation results

Table S6

Functional meta-analysis pathway annotation results

Table S7

Data set for Spearman correlation analysis

References

1. Jiang Y, Chen B, Duan C, Sun B, Yang J, Yang S. 2015. Multigene Editing in the Escherichia coli Genome via the CRISPR-Cas9 System. Applied and Environmental Microbiology 81:2506-2514.

SUPPLEMENTAL FIGURES

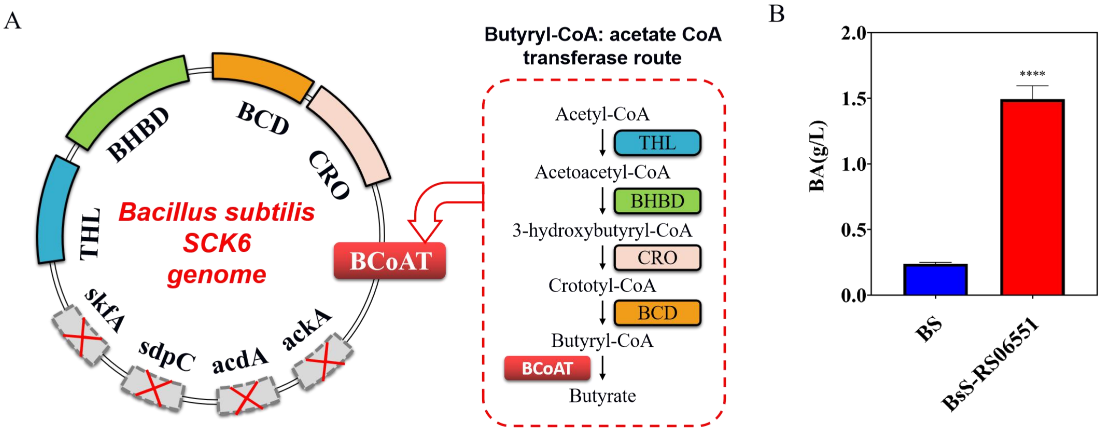

FIG S1

A.Construction strategy of butyrate-producing bacteria. B. Butyric acid production of each strain of engineering bacteria. Abbreviations: THL, thiolase; BHBD, β-hydroxybutyl-CoA dehydrongenase; BCD, butyryl-CoA dehydrogenase; CRO, crotonase; BCoAT, Butyryl-CoA:acetate CoA transferase; skfA, sporulation killing factor; sdpC, sporulation delaying protein C; acdA, acyl-CoA dehydrogenase; acetate kinase; BS, *B. subtilis* SCK6.

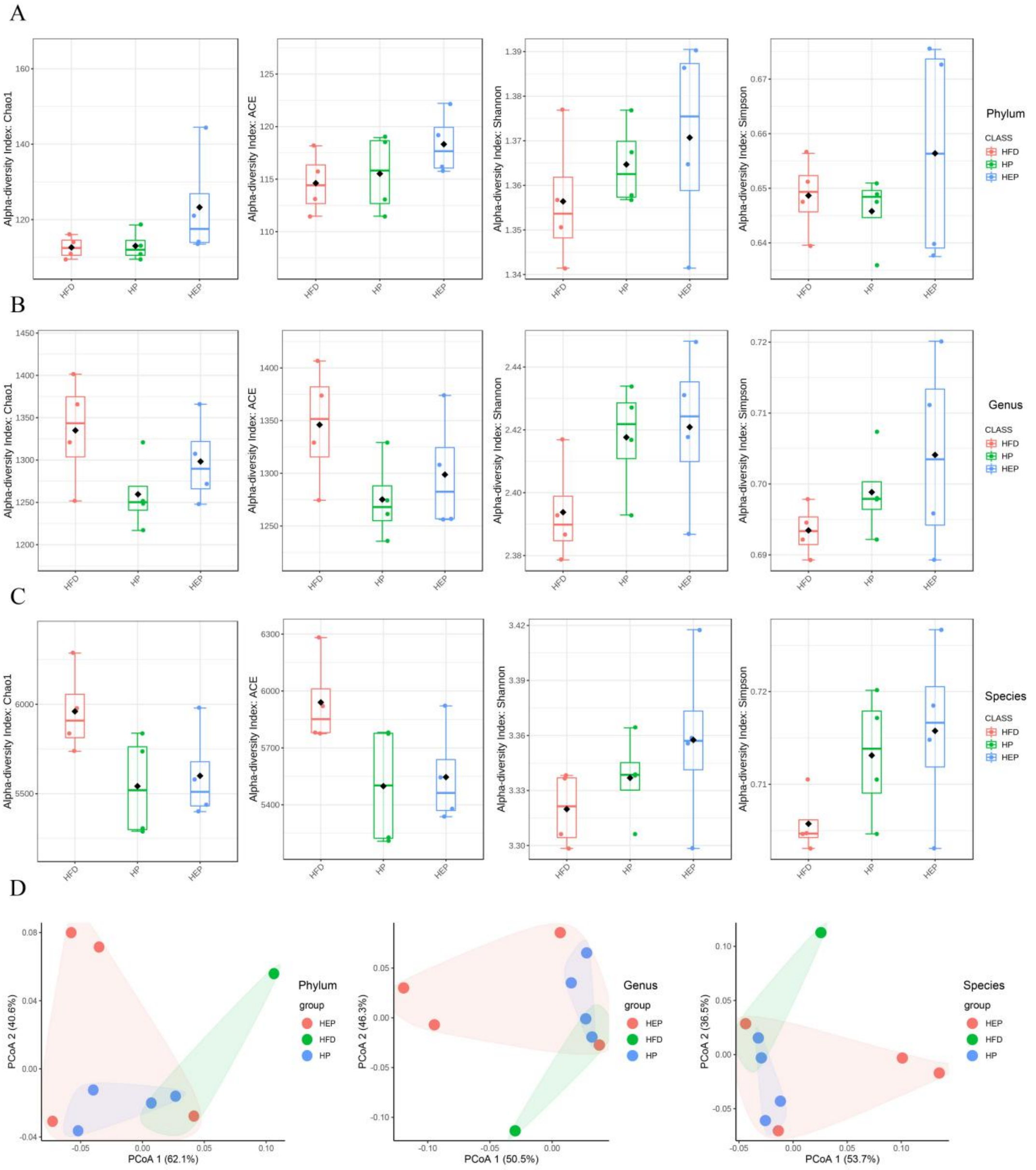

FIG S2

The shift of gut microbiota in different groups of mice according to macrogenomic data. A. Results of alpha diversity analysis at the phylum level. B. Results of alpha diversity analysis at the genus level. C. Results of alpha diversity analysis at the species level. D. PCoA analysis based on the relative abundance of species at the phylum, genus and species levels, respectively.

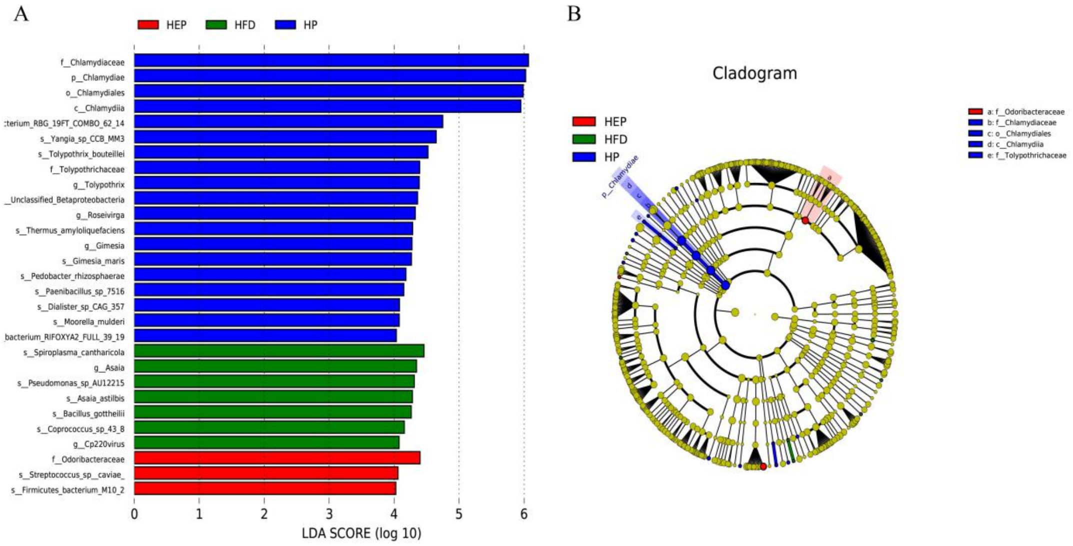

FIG S3

LEfSe analysis of different species between groups. The linear discriminant analysis (LDA) distribution diagram analysis (LAD score >4.0) showed a clear alteration of the microbiota in different individuals. A. Distribution map of LDA values of different species. B. Branch map of different species.

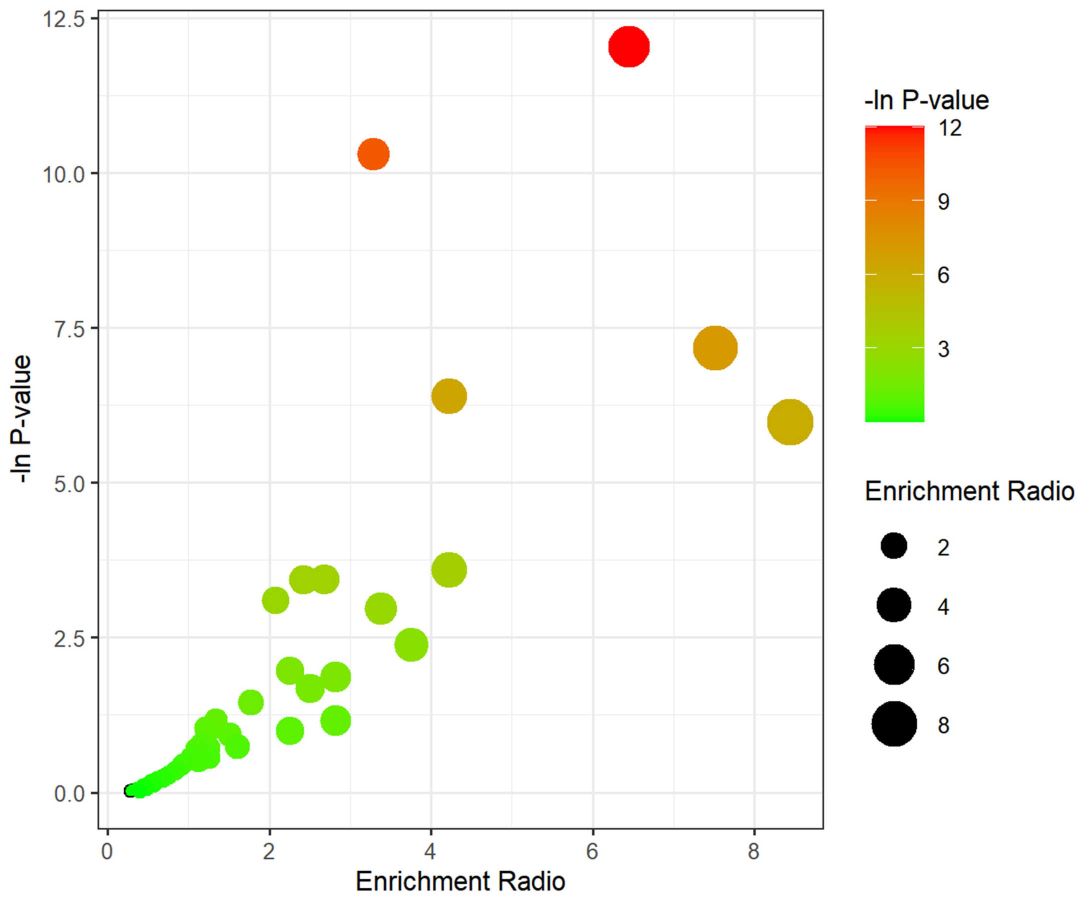

FIG S4

Enrichment analysis pathway annotation results. The plot of functional enrichment analysis for differential metabolites.

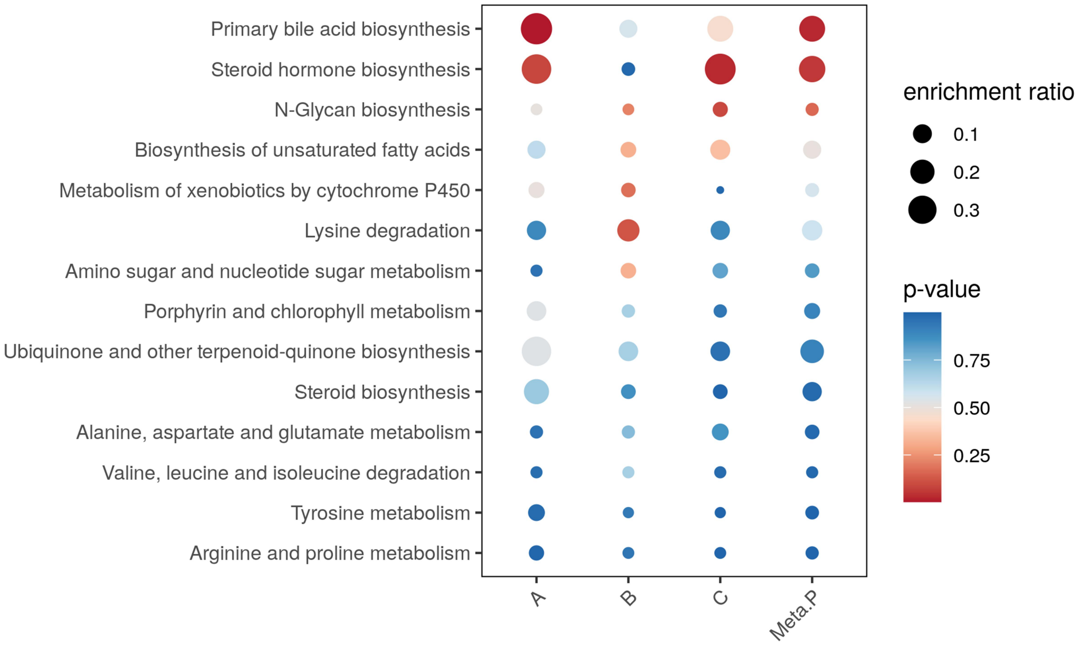

FIG S5

Functional meta-analysis pathway annotation results. The plot of functional meta-analysis for differential metabolites in pairs of 3 groups. A, HFD and HP; B, HFD and HEP; C, HP and HEP.

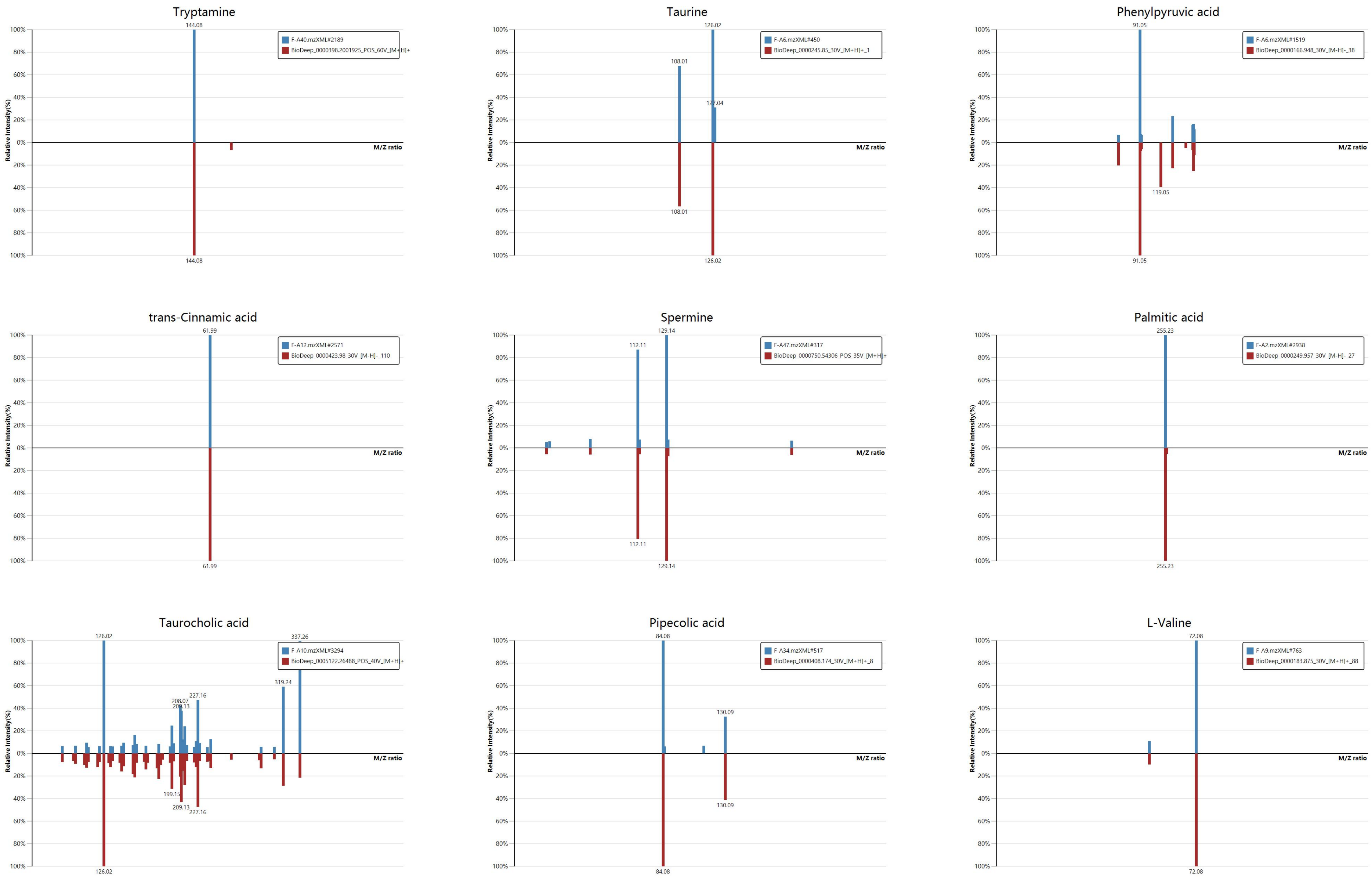
Tryptamine
F-A40.mzXML#2189
BioDeep_0000398.2001925_POS_60V_[M+H]+
Relative Intensity(%)
M/Z ratio
144.08
144.08
Taurine
F-A6.mzXML#450
BioDeep_0000245.85_30V_[M+H]+_1
Relative Intensity(%)
M/Z ratio
126.02
108.01
127.04
108.01
126.02
Phenylpyruvic acid
F-A6.mzXML#1519
BioDeep_0000166.948_30V_[M-H]-_38
Relative Intensity(%)
M/Z ratio
91.05
119.05
91.05
trans-Cinnamic acid
F-A12.mzXML#2571
BioDeep_0000423.98_30V_[M-H]-_110
Relative Intensity(%)
M/Z ratio
61.99
61.99
Spermine
F-A47.mzXML#317
BioDeep_0000750.54306_POS_35V_[M+H]+
Relative Intensity(%)
M/Z ratio
129.14
112.11
112.11
129.14
Palmitic acid
F-A2.mzXML#2938
BioDeep_0000249.957_30V_[M-H]-_27
Relative Intensity(%)
M/Z ratio
255.23
255.23
Taurocholic acid
F-A10.mzXML#3294
BioDeep_0005122.26488_POS_40V_[M+H]+
Relative Intensity(%)
M/Z ratio
126.02
337.26
319.24
227.16
208.07
209.13
199.15
209.13
227.16
126.02
Pipecolic acid
F-A34.mzXML#517
BioDeep_0000408.174_30V_[M+H]+_8
Relative Intensity(%)
M/Z ratio
84.08
130.09
130.09
84.08
L-Valine
F-A9.mzXML#763
BioDeep_0000183.875_30V_[M+H]+_88
Relative Intensity(%)
M/Z ratio
72.08
72.08

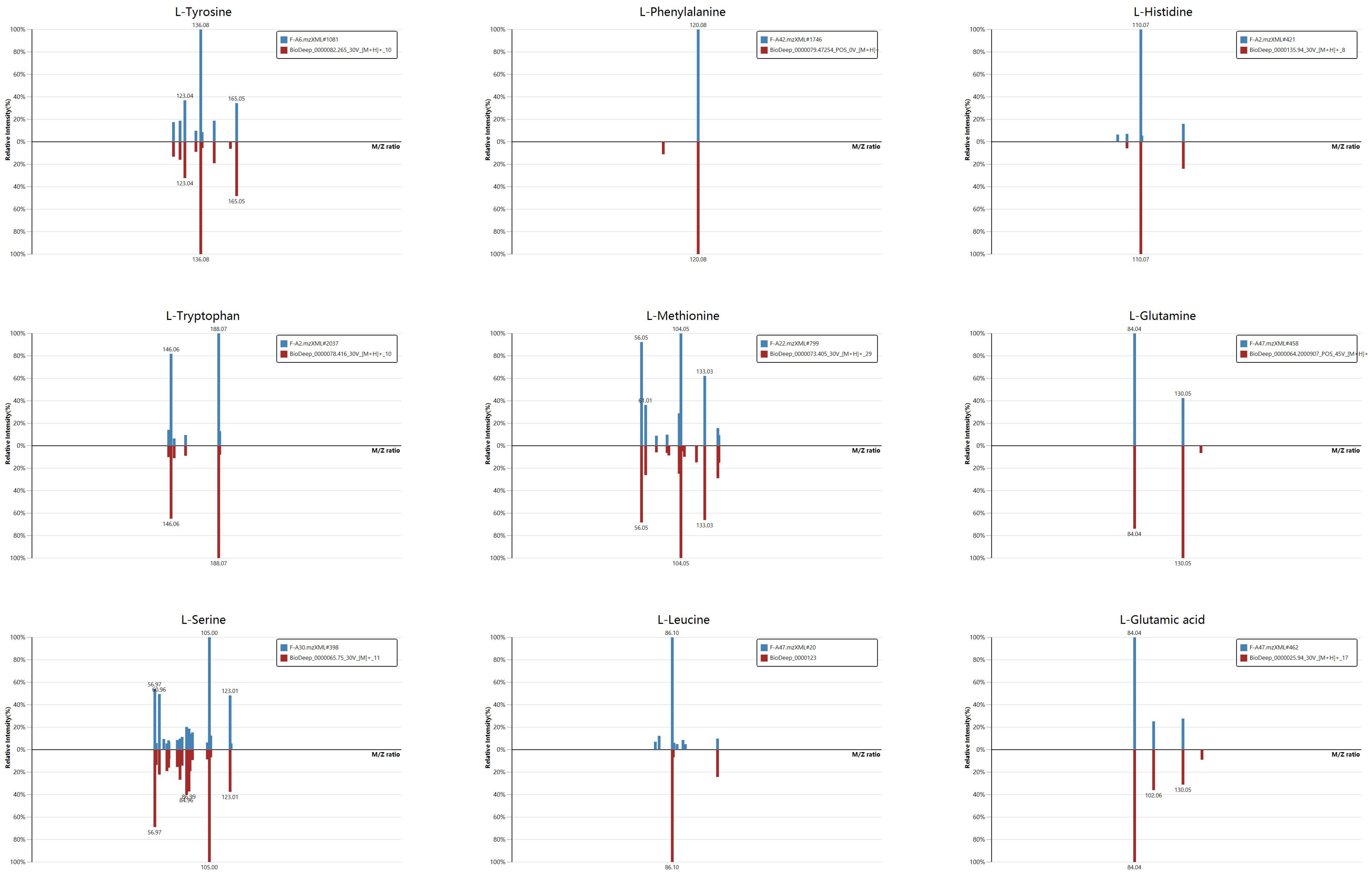
L-Tyrosine
F-A6.mzXML#1081
BioDeep_0000082.265_30V_[M+H]+_10
L-Phenylalanine
F-A42.mzXML#1746
L-Histidine
F-A2.mzXML#421
BioDeep_0000135.94_30V_[M+H]+_8
L-Tryptophan
F-A2.mzXML#2037
BioDeep_0000078.416_30V_[M+H]+_10
L-Methionine
F-A22.mzXML#799
BioDeep_0000073.405_30V_[M+H]+_29
L-Glutamine
F-A47.mzXML#458
L-Serine
F-A30.mzXML#398
BioDeep_0000065.75_30V_[M]+_11
L-Leucine
F-A47.mzXML#20
BioDeep_0000123
L-Glutamic acid
F-A47.mzXML#462
BioDeep_0000025.94_30V_[M+H]+_17
Relative Intensity(%)
M/Z ratio

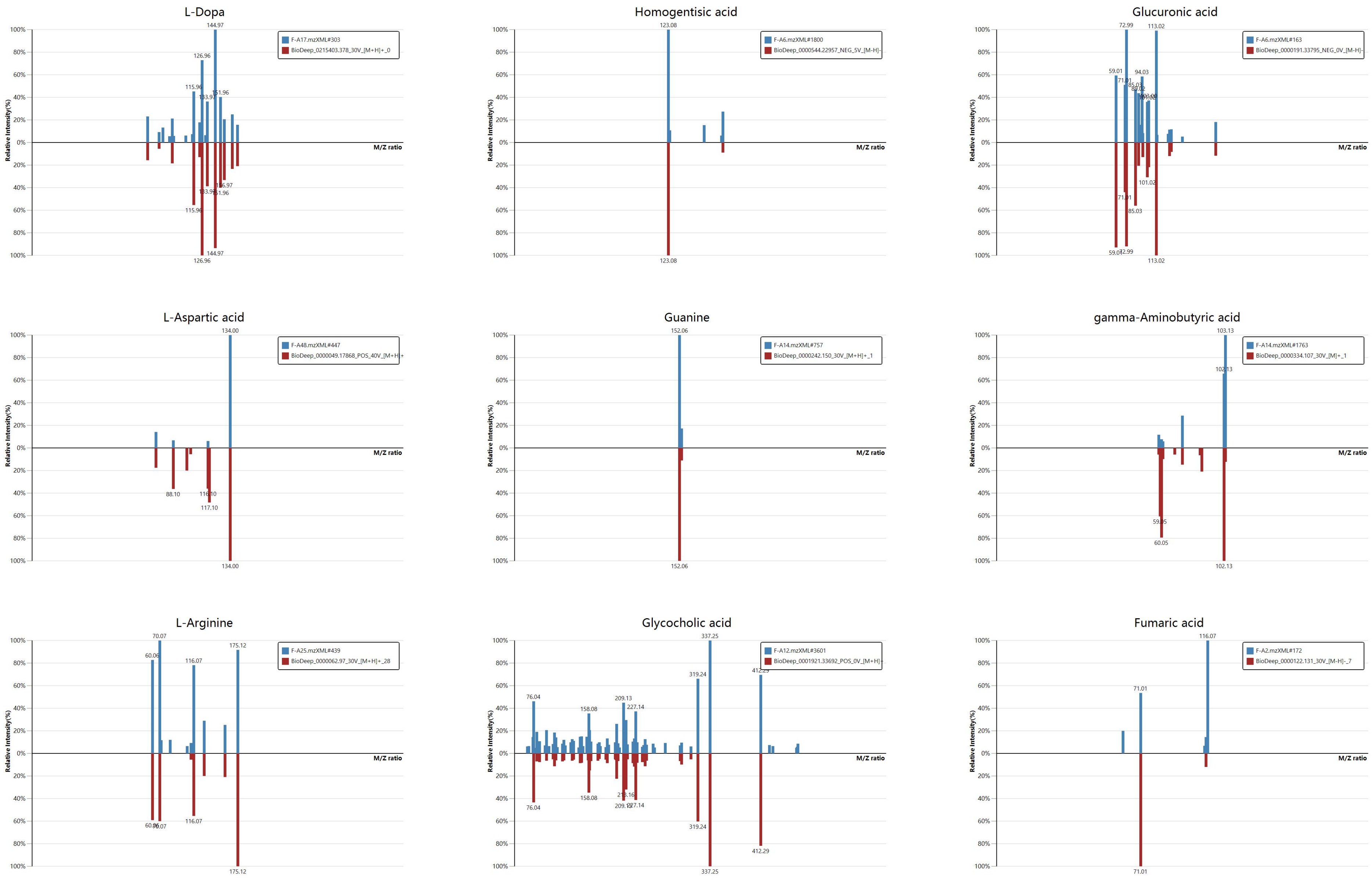
L-Dopa
F-A17.mzXML#303
BioDeep_0215403.378_30V_[M+H]+_0
Homogentisic acid
F-A6.mzXML#1800
BioDeep_0000544.22957_NEG_5V_[M-H]-
Glucuronic acid
F-A6.mzXML#163
BioDeep_0000191.33795_NEG_0V_[M-H]-
L-Aspartic acid
F-A48.mzXML#447
BioDeep_0000049.17868_POS_40V_[M+H]+
Guanine
F-A14.mzXML#757
BioDeep_0000242.150_30V_[M+H]+_1
gamma-Aminobutyric acid
F-A14.mzXML#1763
BioDeep_0000334.107_30V_[M]+_1
L-Arginine
F-A25.mzXML#439
BioDeep_0000062.97_30V_[M+H]+_28
Glycocholic acid
F-A12.mzXML#3601
BioDeep_0001921.33692_POS_0V_[M+H]+
Fumaric acid
F-A2.mzXML#172
BioDeep_0000122.131_30V_[M-H]-_7
Relative Intensity(%)
M/Z ratio

Deoxycholic acid

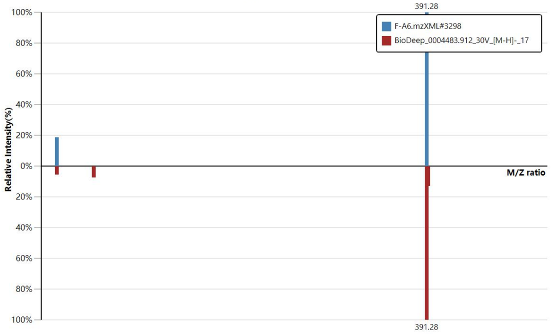

Cholesterol sulfate

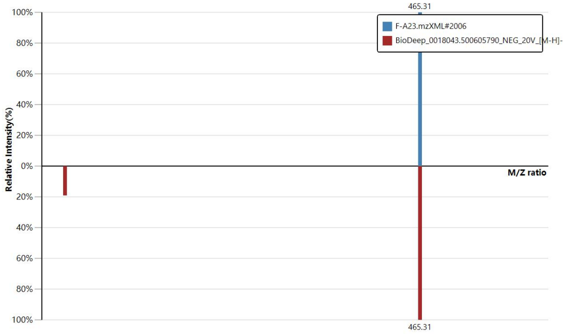

Agmatine

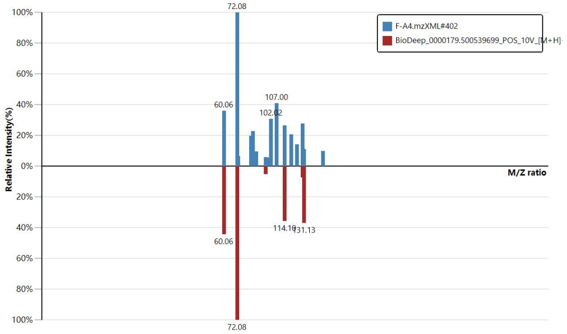

Citrulline

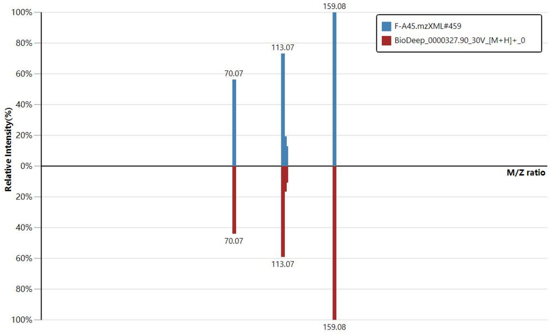

Chenodeoxycholic acid

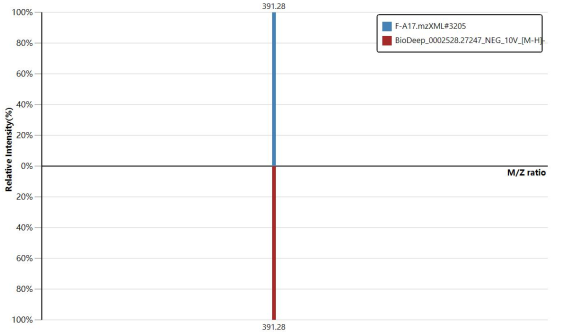

2-Isopropylmalic acid

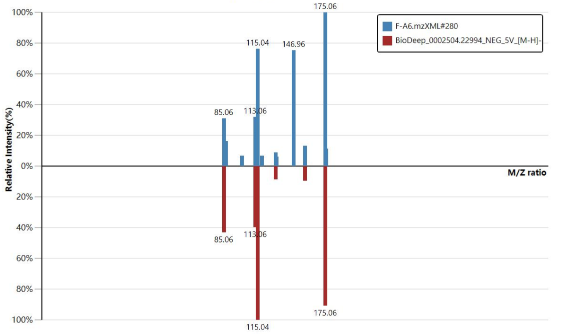

Cholic acid

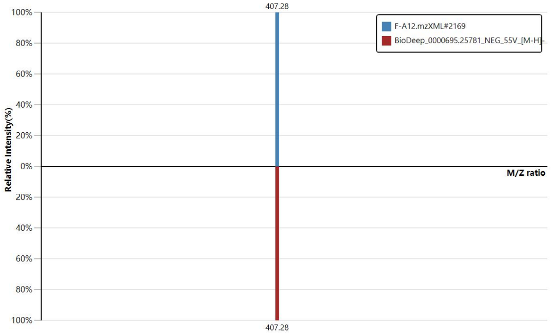

Cadaverine

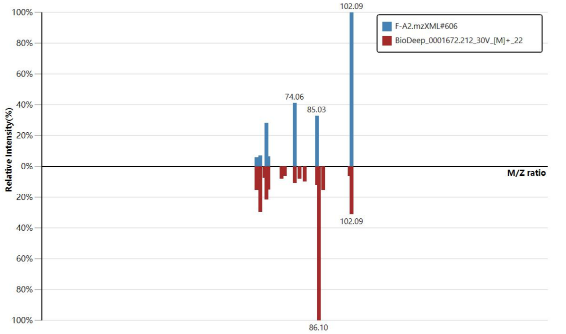

Urocanic acid

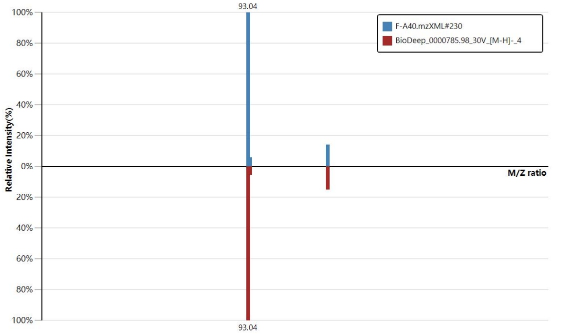

Supplement: SUPPLEMENTAL FILE 1 — Supplemental material. Download SPECTRUM01147-21_Supp_1_seq11.pdf, PDF file, 3.4 MB [file spectrum01147-21_supp_1_seq11.pdf]
